# Editorial: Biological and clinical implications of the mutational landscape in myeloproliferative neoplasms

**DOI:** 10.3389/fonc.2024.1454837

**Published:** 2024-07-16

**Authors:** Giuseppe Gaetano Loscocco, Barbara Mora, Naseema Gangat

**Affiliations:** ^1^ Department of Experimental and Clinical Medicine, CRIMM, Canter of Research and Innovation of Myeloproliferative Neoplasms, Azienda Ospedaliero-Universitaria Careggi, University of Florence, Florence, Italy; ^2^ Division of Hematology, Fondazione IRCCS Cà Granda Ospedale Maggiore Policlinico, Milan, Italy; ^3^ Division of Hematology, Department of Medicine, Mayo Clinic, Rochester, MN, United States

**Keywords:** myeloproliferative neoplasms, *JAK2*, *CALR*, *MPL*, additional mutations, JAK-STAT pathway

Myeloproliferative neoplasms (MPNs) are clonal disorders that include polycythemia vera (PV), essential thrombocythemia (ET), myelofibrosis (MF) both primary (PMF) and secondary to PV and ET, and MPN unclassifiable (MPN-U) (1). Despite differences, all MPNs are characterized by shared clinical and biological features. Clinical complications include Clinical complications include a variety of blood cell alterations, thrombotic and bleeding events, constitutional symptoms caused by pro-inflammatory cytokines, splenomegaly, and risk of progression to acute myeloid leukemia referred to as blast phase MPN (MPN-BP) (2, 3). To date, allogeneic hematopoietic cell transplantation (allo-HSCT) is the only potentially curative treatment but, since it associated with a considerable risk of morbidity and mortality, it should be reserved for selected MF patients (4, 5).

The dysregulation of the JAK/STAT pathway is the hallmark of all MPNs, and it is guided by somatic mutations in driver genes including *JAK2*, *CALR* and *MPL*. *JAK2* mutations are detected in virtually all PV patients and 50-60% of ET and PMF, whereas the remaining are *CALR* (15-25%) and *MPL* mutated (5-10%). Moreover, 10% of ET and PMF patients lack a driver mutation and are referred to as triple-negative (TN) (6). At this point, one of the most challenging questions is: how the same mutations can contribute to the development of different diseases?

There is not a simple answer, but it is reasonable that many factors contribute to phenotypic diversity among PV, ET, and PMF including the variant allele frequency (VAF) of driver mutation (7–10), the type of hematopoietic progenitors targeted by the driver mutation (11), and the host genetic background (12, 13). Moreover, in recent years, high-throughput next-generation sequencing (NGS) approaches have led to identification of other mutations that, in addition to defining the clonality of TN ET and MF (14), contribute to the clonal heterogeneity of MPNs that influence disease phenotype and outcomes (15–20), refractoriness, suboptimal or loss of response to treatments (21–24).

This Research Topic comprises seven papers highlighting the clinical and prognostic implication of mutational landscape in MPNs by using different approaches.

Sobieralski et al. performed a 37-gene targeted NGS analysis on 49 PV and ET patients at two different time points (median time between samples was 104 months). Taking into account the limit of this research the authors documented: i) two coexisting driver mutations in two patients at diagnosis, ii) the most frequently mutated non-driver genes were *TET2* and *DNMT3A*, iii) at second time-point patients with fibrotic progression had a significantly higher VAF of their driver mutation and additional *ASXL1*, *RUNX1*, *ZRSR2*, and *U2AF1* variants were detected, iv) a significant decline of driver mutation VAF was reported in patients treated with hydroxyurea, v) the mean number of variants was higher in patients with longer observation times.

Wang et al. retrospectively analyzed blood counts, cytokines (including IL-1β, IL-2R, IL-6, IL-8, IL-10, TNF-α) and spleen size of 77 MPN patients and 32 patients with idiopathic erythrocytosis and thrombocytosis (IE-IT) with the aim to develop a clinical diagnostic model to discern MPNs and IE/IT. TNF-α levels were significantly higher in MPN, particularly in overt -PMF patients. In addition, median IL-1β levels in PV patients were higher than in other subtypes of MPNs or IE/IT and IL-2R level was also consistently elevated in all subtypes of MPNs. A multivariate logistic regression analysis identified age, platelet count, TNF-α level and spleen size as independent predictive factors for differentiating MPNs and IE/IT.

Olschok et al. described a patient with advanced refractory *JAK2*V617F positive PMF with a complex mutational profile comprising *ASXL1*, *TET2*, *U2AF1*, and *KRAS* mutation that had clinical benefit from treatment with telomerase inhibitor imetelstat for 2.5 years in the MYF2001 phase II trial (NCT02426086). By using patient-derived cells, the authors analyzed the effects of imetelstat on cellular level as well as on hematopoietic cell development. Moreover, the authors demonstrated that imetelstat reduced telomere length and targets JAK/STAT signaling, particularly in *CALR*-mutated cells.

Three case reports highlighted the genetic complexity of MPNs, focusing on different topics as the clonal hematopoiesis of indeterminate potential (CHIP), leukemic progression, and the role of non-canonical driver mutations.

Kjær et al. reported a case of a healthy individual with a *CALR* type-1 mutation detected at a very low VAF (VAF 0.071%) longitudinally monitored over a 12-year period prior to developing a pre-fibrotic MF (VAF>15%). Of interest, in this case, the VAF demonstrated a significant direct correlation with the neutrophil-to-lymphocyte ratio, a marker of chronic inflammation. Generally, *CALR* type-1 mutations confer a favorable prognostic and survival advantage in PMF patients (17, 18, 25, 26). Interestingly, Gurban et al. reported a case of *CALR* type-1 PMF with a concomitant hepatitis C virus (HCV) cirrhosis, who progressed to BP in less than 1 year from diagnosis. Analysis of DNA samples from chronic and leukemic phases by NGS and single-nucleotide polymorphism microarray revealed that the leukemic clone developed from the *CALR*-mutated clone through the acquisition of genetic events in the RAS pathway; moreover, single nucleotide polymorphism (SNP) microarray analysis showed five clinically significant copy number losses, revealing a complex karyotype already in the chronic phase. This case highlights the genetic complexity of MF speculating on the possibility that the interaction between the viral infection, therapy and inflammatory state may have favored the clonal progression. Ligia et al. reported a pediatric PV case with non-canonical *JAK2* G301R variant alongside the *JAK2* V617F mutation, characterized by marked myeloproliferation, splenomegaly and symptoms; the patient was refractory to several treatments and lately disease was well controlled on ropeginterferon alfa-2b. Although functional studies on *JAK2* G301R variant are not available, and its germline origin was not assessed, this case highlights the efficacy of ropeginterferon alfa-2b in managing young symptomatic PV patients, speculating on the potential significance of non-canonical driver variants.

Classically, MPNs are described as Philadelphia (Ph) chromosome negative to be distinguished by Ph chromosome positive (*BCR::ABL1* positive) chronic myeloid leukemia (CML). However, co-occurrence of *BCR::ABL1* and *JAK2* V617F has been reported so far (27). Starting from the presentation of two new cases describing clinical, laboratory, and bone marrow histological findings, Zanelli et al. performed a systematic review of the literature. Overall, 50 papers including 85 cases were analyzed and summarized in three different groups: i) MPNs before CML, ii) CML before MPNs and iii) concomitant MPNs and CML.

In conclusion, this Research Topic underlines the complexity concerning pathogenesis, clinical course, and treatment of MPNs. Additional efforts are needed to better understand the biology of MPNs and identify druggable targets to optimize patient management.

## Author contributions

GGL: Writing – original draft, Writing – review & editing. BM: Writing – original draft, Writing – review & editing. NG: Writing – original draft, Writing – review & editing.
